# Iron Deficiency: Impact on Functional Capacity and Quality of Life in Heart Failure with Preserved Ejection Fraction

**DOI:** 10.3390/jcm9041199

**Published:** 2020-04-22

**Authors:** Alex Alcaide-Aldeano, Alberto Garay, Lídia Alcoberro, Santiago Jiménez-Marrero, Sergi Yun, Marta Tajes, Elena García-Romero, Carles Díez-López, José González-Costello, Gemma Mateus-Porta, Miguel Cainzos-Achirica, Cristina Enjuanes, Josep Comín-Colet, Pedro Moliner

**Affiliations:** 1 University of Barcelona, School of Medicine, 08036 Barcelona, Spain; aalcaial32@alumnes.ub.edu (A.A.-A.); jgcostello@hotmail.com (J.G.-C.); josepcomin@gmail.com (J.C.-C.); 2 Community Heart Failure Unit, Department of Cardiology, Bellvitge University Hospital, L’Hospitalet de Llobregat, 08907 Barcelona, Spain; alberto.garay.melero@gmail.com (A.G.); lidia.alcoberro@gmail.com (L.A.); santijimenezcardio@gmail.com (S.J.-M.); syunvi@gmail.com (S.Y.); mtajes@imim.es (M.T.); cristinaenjuanes@gmail.com (C.E.); 3 Bellvitge Biomedical Research Institute (IDIBELL), L’Hospitalet de Llobregat, 08907 Barcelona, Spain; e.garcia.r@bellvitgehospital.cat (E.G.-R.); carles.diezlopez@gmail.com (C.D.-L.); 4 Department of Internal Medicine, Bellvitge University Hospital, L’Hospitalet de Llobregat, 08907 Barcelona, Spain; 5 Advanced Heart Failure Unit, Department of Cardiology, Bellvitge University Hospital, L’Hospitalet de Llobregat, 08907 Barcelona, Spain; 6 Department of Cardiology, Bellvitge University Hospital, L’Hospitalet de Llobregat, 08907 Barcelona, Spain; gmateus@bellvitgehospital.cat; 7 Johns Hopkins Ciccarone Center for the Prevention of Cardiovascular Disease, Division of Cardiology Johns Hopkins University School of Medicine, Baltimore, MD 21287, USA; miguel.cainzos@gmail.com

**Keywords:** heart failure with preserved ejection fraction, HFpEF, iron deficiency, quality of life, submaximal exercise capacity, 6-min walking test, MLHFQ, 6MWT

## Abstract

The effects of iron deficiency (ID) have been widely studied in heart failure (HF) with reduced ejection fraction. On the other hand, studies in HF with preserved ejection fraction (HFpEF) are few and have included small numbers of participants. The aim of this study was to assess the role that ID plays in functional capacity and quality of life (QoL) in HFpEF while comparing several iron-related biomarkers to be used as potential predictors. ID was defined as ferritin <100 ng/mL or transferrin saturation <20%. Submaximal exercise capacity, measured by the 6-min walking test (6MWT), and QoL, assessed by the Minnesotta Living with Heart Failure Questionnaire (MLHFQ), were compared between iron deficient patients and patients with normal iron status. A total of 447 HFpEF patients were included in the present cross-sectional study, and ID prevalence was 73%. Patients with ID performed worse in the 6MWT compared to patients with normal iron status (ID 271 ± 94 m vs. non-ID 310 ± 108 m, *p* < 0.01). They also scored higher in the MLHFQ, denoting worse QoL (ID 49 ± 22 vs. non-ID 43 ± 23, *p* = 0.01). Regarding iron metabolism biomarkers, serum soluble transferrin receptor (sTfR) was the strongest independent predictor of functional capacity (β = −63, *p* < 0.0001, R^2^ 0.39) and QoL (β = 7.95, *p* < 0.0001, R^2^ 0.14) in multivariate models. This study postulates that ID is associated with worse functional capacity and QoL in HFpEF as well, and that sTfR is the best iron-related biomarker to predict both. Our study also suggests that the effects of ID could differ among HFpEF patients by left ventricular ejection fraction.

## 1. Introduction

Heart failure with preserved ejection fraction (HFpEF), accounts for more than 50% of chronic heart failure (HF) patients [1], with increasing prevalence and comorbidities by ageing. Thus, both population aging and the increasing number of comorbidities have contributed to enlarge the number of patients with this condition over the last years [2]. However, despite its high prevalence and recent research advances, little is known about the pathophysiology and response to medical treatment compared with those patients with heart failure with reduced ejection fraction (HFrEF) [3,4,5,6,7,8]. In fact, medical therapies that have given good results in improving prognosis in patients with HFrEF, such as ACE inhibitors, beta blockers, or sacubitril/valsartan, have not elucidated conclusive results in those with HFpEF so far [9]. Some substudies have suggested that HFpEF patients with left ventricular ejection fraction (LVEF) closer to 50% may respond similarly to medical treatment as patients with ventricular dysfunction, requiring more studies to confirm this fact. This situation leads clinical practice guidelines to recommend being specially aggressive in the diagnosis and management of comorbidities among these patients [6].

Iron deficiency (ID) is a widespread comorbidity among HF patients, with prevalence ranging from 50% in HFrEF to 64% in HFpEF [10,11], and is associated with longer hospital lengths of stay and higher healthcare costs [12,13]. In HFrEF, ID has been thoroughly studied, and there is strong evidence showing an association between ID and worse exercise capacity and quality of life, and treatment with intravenous ferric carboxymaltose has been tested and approved with demonstrated clinical benefit [14,15,16,17,18,19]. However, there is less evidence regarding the same association in HFpEF [20], with evidence consisting of small observational and heterogenous studies, some of which suggest that ID may be associated with reduced functional capacity [21] and worse quality of life [22].

Therefore, it is necessary to outline exhaustively this highly prevalent ID in HFpEF in order to characterize its clinical repercussion. It could also be useful to explore whether this potential effect behaves similarly across the LVEF spectrum within HFpEF.

The aim of this study was to assess the association of ID in functional capacity and quality of life among HFpEF patients while comparing several iron-related parameters to be used as potential predictors and, finally, to explore possible differences according to LVEF.

## 2. Methods

### 2.1. Study Design and Population

The DAMOCLES study (Definition of the neuro-hormonal Activation, Myocardial function, genOmic expression and CLinical outcomes in hEart failure patientS) was a single-center, observational, prospective cohort study of 1236 consecutive patients diagnosed with chronic HF from 2004 to 2013. For the present secondary analysis, only baseline data from the study was used, acquiring a cross-sectional design. The methodology of the study has been previously published by our group [19]. Briefly, for inclusion, patients had to be diagnosed with chronic HF according to the European Society of Cardiology diagnostic criteria, have at least one recent acute chronic HF decompensation requiring intravenous diuretic therapy (either hospitalized or in the day care hospital), and had to be in stable condition at the time of study entry. Exclusion criteria were haemoglobin (Hb) levels <8.5 g/dL, significant primary valvular disease, clinical signs of fluid overload, restrictive or hypertrophic cardiomyopathy, pericardial disease, active malignancy, and chronic liver disease. The study was approved by the local ethics committee for clinical research (2004/1788/I) and was conducted in accordance with the principles of the Declaration of Helsinki. All patients gave written informed consent before study entry.

### 2.2. Study Definitions

Iron deficiency was assessed using the Kidney Disease Outcomes Quality Initiative (KDOQI) criteria [23], which categorizes ID as ferritin <100 ng/mL and/or transferrin saturation (TSAT) <20%. Functional capacity was measured by the 6-min walking test (6MWT), determining the submaximal exercise capacity in those who were able to walk. It consists of walking the longest distance possible during 6 min over a 30-m straight line loop. Quality of life was assessed with the Minnesota Living with Heart Failure Questionnaire (MLHFQ). This test covers physical, emotional, and social issues with 21 items, each ranging from 0 to 5, with higher scores denoting worse quality of life. Anemia was defined using the World Health Organization (WHO) guidelines (Hb < 130 g/L in men and Hb < 120 g/L in non-pregnant women). Iron metabolism laboratory parameters determined were Hb, serum iron, transferrin, transferrin saturation, ferritin, ferritin index, and soluble transferrin receptor (sTfR). For the present analysis, only patients with ejection fraction equal or higher than 50% were included, as assessed by echocardiography.

### 2.3. Statistical Analysis

Using the baseline data from the DAMOCLES cohort, a cross-sectional analysis was performed. Demographic and clinical characteristics, as well as laboratory test results, were summarized using basic descriptive statistics, both overall and categorized by iron deficiency. For quantitative variables, arithmetic mean and standard deviation or median and interquartile ranges were reported, and *p* values were obtained using the *t*-test or Mann-Whitney *U* test. For qualitative variables, number and percentages within specified groups were calculated using the χ^2^ test for the p values.

Univariate and multivariate linear regression models were performed with several iron-related parameters in order to predict 6MWT results and MLHFQ scores, which formed a normal distribution. Logarithmic transformation was used to fit skewed continuous variables, such as ferritin, serum iron, and sTfR, into normal distributions when necessary. Unadjusted models were used in order to clarify the effect of each independent parameter, whereas multivariate ones increased relevance and clinical context, as well as significance and determination coefficient (R^2^). Auto-adjusted generalized additive models (GAMs) were used to assess nonlinear associations between (sTfR) and exercise capacity and quality of life depending on LVEF. Finally, to explore possible differences in ID effect according to LVEF in HFpEF patients, we repeated the different analyses based on whether the LVEF was >60% or ≤60%.

All *p* values reported were constructed with a type-I error alpha level of 5% with no adjustments for multiplicity and *p* values < 0.05 were considered statistically significant. Data was analyzed using Rstudio 3.0.1. R: A language and environment for statistical computing (R Foundation for Statistical Computing, Vienna, Austria).

## 3. Results

### 3.1. Baseline Characteristics

From the total 1236 chronic HF patients included in the DAMOCLES study, only those with EF ≥ 50% and with iron-related parameters tested were selected (*N* = 447). Patients were subdivided in two groups according to the presence of ID (*N* = 325) or not (*N* = 122). The baseline characteristics of the study sample, both overall and according to ID, are shown in Table 1. Mean age was 76 ± 9 years, LVEF was 62 ± 8%, patients were 59% female, and ID prevalence was markedly high, accounting for 73% of patients.

Patients with ID were more frequently female, with higher rates of anemia and diabetes, and higher levels of C-reactive protein. There were almost no differences in medical treatment between groups.

### 3.2. Functional Capacity and Iron Deficiency

**Figure 1:**
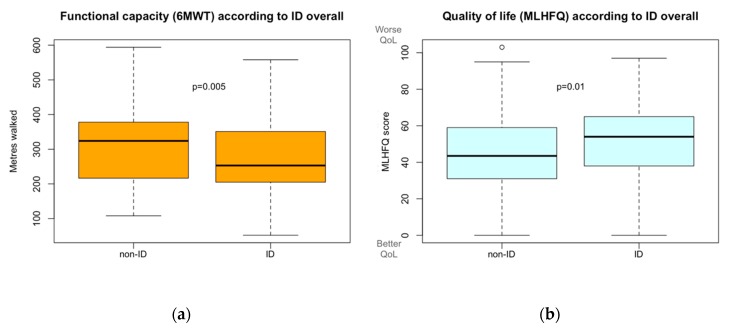
(**a**) Functional capacity, measured with 6-min walking test (6MWT), according to iron deficiency. (**b**) Quality of life, measured with Minnesota Living with Heart Failure Questionnaire (MLHFQ), according to iron deficiency.

A total of 282 patients were able to undergo the 6MWT, 202 in the ID group and 80 in the non-ID group, whereas 107 and 36 were not, respectively. As summarized in Figure 1 and Table 2, the distance walked by patients with ID was lower than that walked by patients with normal iron status (ID patients 271 ± 94 m vs. non-ID 310 ± 109 m; *p* = 0.005). Furthermore, the number of patients who had to discontinue the test before its completion was greater in the ID group compared to patients without ID. Interestingly, this worse functional capacity present in patients with HFpEF and ID was also observed in those patients without anemia (*p* < 0.03).

### 3.3. Quality of Life and Iron Deficiency

Using the MLHFQ to assess quality of life, statistically significant differences were found between ID and non-ID patients. As seen in Figure 1 and Table 2, the ID group scored six points higher in the global score than non-ID patients, (49.4 ± 22 vs. 43.1 ± 23 points respectively; *p* = 0.01), reflecting worse quality of life.

When we analyzed these results according to the different dimensions evaluated in the MLHFQ, physical, as well as social and personal dimensions, were the most affected (physical: 28.4 ± 12 vs. 25.4 ± 13, *p* = 0.025; social and personal: 8.5 ± 6.7 vs. 6.9 ± 6.3, *p* = 0.018), although the remaining two dimensions were close to the significance threshold. Results subdivided by items are available in Table S1. Resting, housekeeping, sleeping, socializing, hobbies, and depression were the most impaired factors in patients with HFpEF and ID compared with patients with HFpEF and normal iron status. Opposite to functional capacity, no statistically significant differences were found in patients with ID vs. non-ID when we analyzed quality of life in non-anemic patients only.

### 3.4. Linear Regression Predictive Models

Several iron-related parameters and definitions were tested in univariate and multivariate models to predict 6MWT results and MLHFQ scores. These models were adjusted by age, sex, NYHA functional class, and LVEF, since these were the variables that had a statistically significant impact in the model when including all Table 1 variables.

The definition of ID we used (ferritin <100 ng/mL or TSAT <20%) was independently associated with worse results in 6MWT (β = −29.7; R^2^ = 0.35; *p* < 0.01) and higher scores in MLHFQ, denoting worse quality of life (β = 4.9; R^2^ = 0.11; *p* < 0.04) in patients with HFpEF. On the other hand, the association of the classic definition of the FAIR-HF study [24] (ferritin <100 ng/mL or 100–300 ng/mL with TSAT <20%) with exercise capacity was weaker (β = −23.5; R^2^ = 0.35; *p* < 0.03) and was not significantly associated with worse quality of life (*p* = 0.21). This worse predictive capacity of the FAIR-HF definition is consistent with biomarkers univariate results that we obtained, since ferritin was not associated with functional capacity or quality of life, whereas TSAT did in both.

Our results showed that sTfR was the best iron-related biomarker since it was the only laboratory parameter that was significantly associated with both worse functional capacity (β = −27.1; R^2^ = 0.36; *p* < 0.04) and poorer quality of life (β = 7.9; R^2^ = 0.15; *p* < 0.001) in multivariate models. Detailed results are shown in Table 3.

### 3.5. Differences within HFpEF according to LVEF

Additionally, in order to elucidate possible differences along EF range within HFpEF, our study population was further subdivided in two groups, LVEF >60% (*N* = 220) and LVEF ≤60% (*N* = 227). Baseline characteristics according to these two groups are shown in Table S2. Compared with the LVEF >60% group, those patients with LVEF ≤60% were more frequently male (50% vs. 31%; *p* < 0.01) and had more ischemic heart disease (27% vs. 17%; *p* = 0.01). We found no differences regarding age, comorbidities, or functional class.

**Figure 2:**
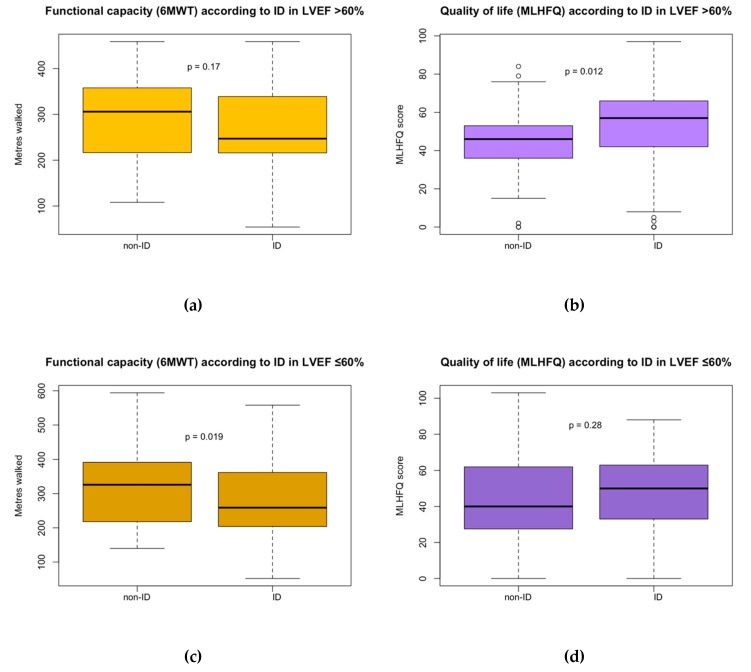
(**a**) Functional capacity (measured with 6MWT) according to iron deficiency in HFpEF with LVEF >60%. (**b**) Quality of life (measured with MLHFQ) according to iron deficiency in HFpEF with LVEF >60%. (**c**) Functional capacity (measured with 6MWT) according to iron deficiency in HFpEF with LVEF ≤60%. (**d**) Quality of life (measured with MLHFQ) according to iron deficiency in HFpEF with LVEF ≤60%.

Interestingly, we found differences regarding the association of ID in HFpEF with both functional capacity and quality of life according to the two groups of LVEF (Figure 2 and Table 4). The association we had previously found between ID and poorer functional capacity was also observed in multivariate models in the subgroup of patients with HFpEF and LVEF 50–60% (β= −37.3; R^2^ = 0.33; *p* < 0.03). However, we did not find a significant association in the group of patients with LVEF >60% (*p* = 0.20). In contrast, the association of ID with worse quality of life was found in the group of patients with LVEF > 60% (β = 6.8; R^2^ = 0.15; *p* < 0.04) but not in patients with LVEF 50–60% (*p* = 0.41).

**Figure 3:**
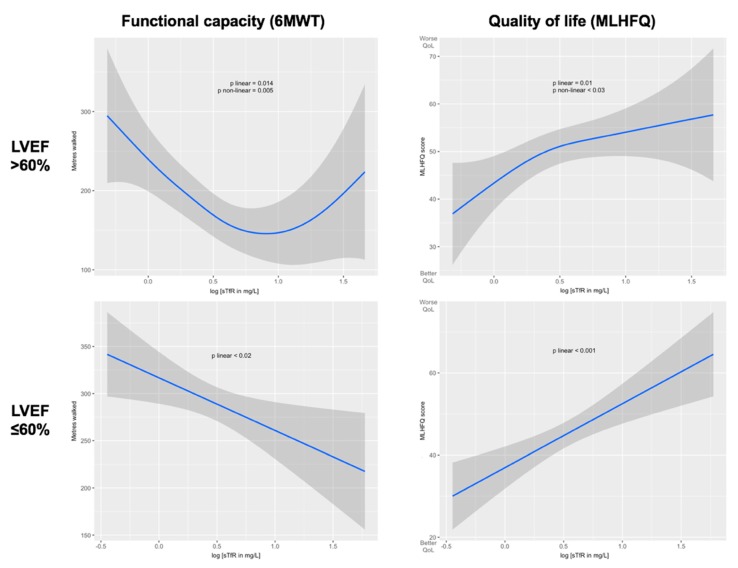
Unadjusted associations between sTfR (log-transformed) and functional capacity and quality of life, separated by LVEF >60% or ≤60%, and calculated using generalized additive models (GAM). 6MWT (6-min walking test), LVEF (left ventricular ejection fraction), MLHFQ (Minnesota Living with Heart Failure Questionnaire), sTfR (serum soluble transferrin receptor).

Finally, sTfR, which showed to be the best biomarker of iron status to predict worse exercise capacity and quality of life in the whole cohort of HFpEF, did not show a significant association with either in the subgroup of patients with LVEF >60% in multivariate linear regression models. Again, we found a different response in the association of ID depending on the LVEF in HFpEF patients. We explored these different associations performing generalized additive models (GAM) models, as seen in Figure 3, where we confirmed linear associations in LVEF ≤60% with worse functional capacity (*p* < 0.02) and worse quality of life (*p* < 0.001). In LVEF >60%, however, we found nonlinear associations with functional capacity (*p* nonlinear < 0.005) and quality of life (*p* nonlinear < 0.03).

## 4. Discussion

This study has shown an association between ID and both worse functional capacity and quality of life in HFpEF patients. To the best of our knowledge, this is the largest study on this field, highlighting sTfR as the best predictor biomarker for both in HFpEF for the first time. Furthermore, our study postulates that the effects of ID in patients with HFpEF may be different according to their LVEF. This could support recent studies suggesting patients with HFpEF are a heterogeneous group. Another aspect to highlight is the high prevalence of ID in HFpEF (73%), based on the KDOQI definition of ID, which was superior to the current ID definition based on the FAIR-HF study for the prediction of worse functional capacity and quality of life in patients with HFpEF.

### 4.1. Functional Capacity Impairment and Quality of Life Worsening by Iron Deficiency

Iron is an essential component for the proper function of the mitochondria and, therefore, ID leads to an energy metabolism alteration that is not limited to cardiomyocytes, where it causes cardiac enlargement, ventricular hypertrophy, diastolic dysfunction, and progressive cardiac fibrosis, but also has effects on the striated muscle cells of the musculoskeletal system [25]. Thus, the exercise intolerance observed in patients with HFpEF is probably multifactorial, derived from the interaction of pulmonary, cardiac, hematological, and skeletal muscle impairments [26,27,28,29].

As summarized recently by Beale et al. [20], several related investigations in smaller studies regarding ID in HFpEF had been published within the last years, and most of these studies have pointed toward a common direction. Nuñez et al. [21] studied a cohort of 40 patients with advanced HFpEF and found that ID was associated with poorer exercise capacity. Similar results were obtained by Martens et al. in 59 patients with HFpEF [10]. In both studies, an impaired functional capacity measured by peak VO_2_ was observed among ID patients. Additionally, the role of TSAT and serum ferritin as predictors were highlighted in the former, although no information was provided about sTfR. 

Regarding quality of life, in a 110 HFpEF patients cohort, Bekfani et al. [22] suggested that, contrary to our study, there were not significant differences in quality of life measured with EQ5D between ID and non-ID patients. Nevertheless, there was a trend toward the significant threshold. They also assessed functional capacity differences measured with 6MWT. Low TSAT was associated with worse quality of life, in accordance with our results. However, they also found that low serum ferritin was associated with worse functional capacity, although our findings do not support this association. In our study, several biomarkers of iron status were evaluated. Hepcidin, a main regulator of iron uptake and release, was not evaluated due to the lack of that parameter, although it has been associated with ID [30,31]. Furthermore, differences between HFrEF and HFpEF have been suggested [32]. TSAT was associated with worse functional capacity, while sTfR showed the highest performance as a predictor of functional capacity and also quality of life. sTfR is a biomarker that provides information on tissue iron demand and because it is insensitive to inflammation, it can detect anemia in patients with preexisting inflammatory states, which is particularly useful in distinguishing between anemia of chronic disease and anemias caused by lack of iron intake [33,34,35]. Previous studies of our group had already shown its ability to predict effort capacity and quality of life in patients with chronic HF globally [18], and this paper studies its impact and predictive value specifically in HFpEF compared with other iron metabolism biomarkers and explores differences along the HFpEF LVEF range.

### 4.2. Iron Deficiency in HFpEF according to LVEF

Recently, the use of sacubitril/valsartan was evaluated in patients with HFpEF in the PARAGON-HF study [9]. No significant improvement was seen at the primary endpoint, but subsequent subanalyses suggested that those patients with HFpEF and LVEF closer to 50% may have behaved differently and might benefit from this treatment. Future studies should confirm this possibility, but these results suggest that there are differences within HFpEF according to LVEF. In our cohort, we decided to explore if there were also differences in the consequences of ID based on LVEF. ID was associated with worse results in the 6MWT at the expense of patients with LVEF between 50% and 60%, while the association with worse scores in the quality of life test was due to the subgroup of patients with LVEF above 60%. We even found differences in the predictive capacity of sTfR in multivariate linear regression models, being significant for the group with LVEF 50–60%, but not in patients with LVEF >60%, since the association in this subgroup was nonlinear. These exploratory analyses suggest that HFpEF is a heterogeneous group, but further research is needed in order to confirm these results and expand the knowledge about the reason for these differences.

### 4.3. Clinical Implications

Once the association of ID to worse exercise capacity and poorer quality of life was assessed in HFpEF patients, the next step would be a randomized clinical trial testing iron-depletive treatments to mitigate the differences.

As we explained before, HFrEF studies have shown a benefit receiving intravenous (IV) ferric carboxymaltose, with current guidelines supporting its consideration in symptomatic patients with ID and HFrEF, leaving oral iron therapy as ineffective for replenishment of iron stores and improvement of clinical status [6,11,17]. However, there is not enough evidence regarding HFpEF yet. There are currently two ongoing randomized clinical trials, the FAIR-HFpEF (NCT03074591) [36] and the PREFER-HF (NCT03833336) [37], the results of which are eagerly awaited and will be showcased in the upcoming years.

### 4.4. Limitations of the Study

Some limitations must be taken into account. First, given its single center cross-sectional design, the single determinations of iron profile, functional capacity, and quality of life did not allow us to provide information about their evolution. Second, the inclusion criteria required an exacerbation in the last 12 months, and this could have therefore favored advanced-stage HFpEF patients, as well as diminished the number of 6MWT-eligible patients. Third, the use of 6MWT to measure functional capacity instead of peak VO_2_ may have masked the maximal exertion performance. Additionally, existing comorbidities in our study population could condition 6MWT results. However, excluding anemia, which has been analyzed and reported subsequently, the remaining comorbidities affected both groups equally, thus outshining its effects.

## 5. Conclusions

In this cohort, we determined that ID was highly prevalent and was associated with worse functional capacity and quality of life in HFpEF patients. After the assessment of several iron-related biomarkers, sTfR showed the best performance to be used as a potential predictor of both. Exploratory analyses suggest possible differences in the association of ID according to LVEF in HFpEF patients, although further research is needed to confirm and explain these findings.

## Figures and Tables

**Table 1:** Baseline characteristics of HFpEF patients according to ID.

	Overall *N* = 447	Iron Deficiency *N* = 325 (73%)	No iron Deficiency *N* = 122 (27%)	*p* Value
Age (years)	75.7 ± 9.2	75.7 ± 8.7	75.9 ± 10.3	0.84
Female sex	264 (59.1)	202 (62.2)	62 (50.8)	0.039
BMI (kg/m^2^)	29.1 ± 6.2	29.4 ± 6.3	28.5 ± 6.0	0.19
Systolic BP (mmHg)	128.4 ± 21.8	127.9 ± 20.6	131.6 ± 24.4	0.10
Heart rate (bpm)	73.7 ± 14.4	74.0 ± 14.6	73.4 ± 14.1	0.71
LVEF (%)	62.1 ± 7.8	62.8 ± 7.9	60.7 ± 7.3	0.011
Ischemic etiology of CHF	100 (22.4)	77 (23.7)	23 (18.9)	0.33
NYHA functional class				
I	47 (10.5)	31 (9.5)	16 (13.1)	0.36
II	193 (43.2)	143 (44.0)	50 (40.9)	0.64
III	166 (37.1)	122 (37.5)	44 (36.1)	0.86
IV	38 (8.5)	27 (8.3)	11 (9.0)	0.96
Comorbidities				
Hypertension	391 (87.5)	290 (89.2)	101 (82.8)	0.09
COPD	93 (20.8)	67 (20.6)	26 (21.3)	0.98
Diabetes mellitus	221 (49.4)	175 (53.8)	46 (37.7)	0.003
Chronic kidney disease	282 (63.1)	208 (64.0)	74 (60.7)	0.59
Anemia	249 (55.7)	195 (60.0)	54 (44.3)	0.004
Dependency	142 (31.8)	112 (34.6)	30 (24.6)	0.16
Medications				
ACEIs or ARBs	300 (67.1)	212 (65.2)	88 (72.1)	0.20
Beta-blockers	352 (78.7)	261 (80.3)	91 (74.6)	0.24
MRAs	50 (11.2)	34 (10.5)	16 (13.1)	0.61
Digoxin	71 (15.9)	46 (14.2)	25 (20.5)	0.14
Loop diuretics	411 (91.9)	293 (90.2)	118 (96.7)	0.038
Statins	249 (55.7)	187 (57.5)	62 (50.8)	0.24
Antiplatelets	155 (34.7)	115 (35.4)	40 (32.8)	0.69
Anticoagulants	260 (58.2)	186 (57.2)	74 (60.7)	0.58
Laboratory parameters				
Hemoglobin (g/dL)	12.26 ± 1.89	11.91 ± 1.76	12.86 ± 2.03	<0.001
eGFR (mL/min/1.73 m^2^)	55.29 ± 23.78	54.08 ± 23.76	58.06 ± 23.33	0.11
Ferritin (ng/mL)	141 (70.5–285.0)	99 (55.0–219.0)	274.5 (178.75–423)	<0.001
Transferrin (mg/dL)	248.02 ± 51.58	256.10 ± 53.31	226.57 ± 39.49	<0.001
Serum iron (pg/dL)	61.80 ± 32.90	50.83 ± 24.81	91.02 ± 34.01	<0.001
TSAT (%)	18.61 ± 11.17	14.42 ± 6.18	29.74 ± 13.60	<0.001
Ferritin index	0.95 ± 0.71	1.06 ± 0.78	0.62 ± 0.27	<0.001
sTfR (mg/L)	1.92 ± 1.58	2.07 ± 1.79	1.51 ± 0.66	<0.001
NT-proBNP (pg/mL)	1284 (678–2876)	1304 (685.5–2827.5)	1108.5 (642–3303.5)	0.50
C-reactive protein (mg/dL)	1.66 ± 2.35	1.82 ± 2.57	1.23 ± 1.58	0.004

Data presented as mean ± SD, N (%) or median (interquartile range). ACEI (angiotensin converting enzyme inhibitor), ARB (angiotensin receptor blocker), BMI (body mass index), BP (blood pressure), CHF (chronic heart failure), COPD (chronic obstructive pulmonary disease), eGFR (estimated glomerular filtration rate), HFpEF (heart failure with preserved ejection fraction), ID (iron deficiency), LVEF (left ventricular ejection fraction), MRA (mineralocorticoid receptor antagonist), NT-proBNP (N-terminal pro-B type natriuretic peptide), NYHA (New York Heart Association), sTfR (serum soluble transferrin receptor), TSAT (transferrin saturation). Dependency defined as Barthel test score <90 points.

**Table 2:** Functional capacity and quality of life according to iron deficiency.

	Iron Deficiency	No Iron Deficiency	*p* Value
**6-min walking test (6MWT)**	*N* = 309	*N* = 116	
Meters walked			
Overall, *N* = 202 and *N* = 80	270.7 ± 94.0	310.0 ± 108.9	0.005
Non-anemics, *N* = 94 and *N* = 50	286.8 ± 90.2	327.9 ± 112.1	0.028
N of subjects that walked >300 m	72 (35.6)	47 (58.8)	<0.001
N of subjects unable to complete the test	59 (29.2)	13 (16.3)	0.041
N of subjects that developed symptoms	77 (38.1)	22 (27.5)	0.12
N of subjects unable to undergo the test	107 (34.6)	36 (31.0)	0.56
**Minnesota living with HF questionnaire (MLHFQ)**	*N* = 325	*N* = 122	
Overall summary score (min: 0, max: 105)			
Overall, *N* = 325 and *N* = 122	49.4 ± 22.44	43.1 ± 23.2	0.01
Non-anemics, *N* = 130 & *N* = 68	46.4 ± 26.4	43.8 ± 24.0	0.46
Composite scores			
Physical dimension (min: 0, max: 40) (sum of items: 2, 3, 4, 5, 6, 7, 12, 13)	28.4 ± 12.1	25.4 ± 12.7	0.025
Emotional dimension (min: 0, max: 25) (sum of items: 17, 18, 19, 20, 21)	8.2 ± 6.0	6.9 ± 6.4	0.06
Socioeconomic dimension (min: 0, max: 10) (sum of items: 14, 15)	4.0 ± 2.7	3.5 ± 2.7	0.09
Social and personal dimension (min: 0, max: 25) (sum of items: 7, 8, 9, 10, 11)	8.5 ± 6.7	6.9 ± 6.3	0.018

Data presented as mean ± SD or *N* (%).

**Table 3:** Univariate and multivariate linear regression models for 6MWT and MLHFQ.

	Univariate Models			Multivariate Models		
	β Coef.	*p* Value	Adj. R^2^	β Coef.	*p* Value	Adj. R^2^
**6-Min Walking Test (6MWT)**						
vs. iron deficiency (KDOQI)	−39.77	0.003	0.03	−29.66	0.007	0.35
vs. iron deficiency (FAIR-HF)	−37.23	0.002	0.03	−23.35	0.021	0.35
vs. anemia (WHO)	−40.90	<0.001	0.04	−21.10	0.035	0.34
vs. TSAT < 20%	−36.07	0.004	0.03	−28.42	0.006	0.35
vs. sTfR (log)	−29.51	0.056	0.01	−27.09	0.035	0.36
vs. ferritin (log)	3.71	0.55	<0.01	−4.99	0.34	0.34
vs. serum iron (log)	29.06	0.012	0.05	9.77	0.32	0.34
**Minnesota Living with Heart Failure Questionnaire (MLHFQ)**						
vs. iron deficiency (KDOQI)	6.31	0.009	0.01	4.80	0.039	0.11
vs. iron deficiency (FAIR−HF)	4.76	0.032	<0.01	2.69	0.21	0.10
vs. anemia (WHO)	3.78	0.08	<0.01	1.42	0.50	0.10
vs. TSAT < 20%	4.99	0.029	<0.01	3.32	0.13	0.10
vs. sTfR (log)	12.96	<0.001	0.05	7.95	<0.001	0.15
vs. ferritin (log)	−0.58	0.61	<0.01	0.64	0.56	0.10
vs. serum iron (log)	−3.76	0.08	<0.01	−1.50	0.47	0.10

6MWT (6-Min Walking Test), adj. (adjusted), coef. (coefficient), FAIR-HF (Ferinject Assessment in patients with IRon deficiency and chronic Heart Failure study), KDOQI (Kidney Disease Outcomes Quality Initiative), LVEF (left ventricular ejection fraction), MLHFQ (Minnesota Living with Heart Failure Questionnaire), NYHA (New York Heart Association), sTfR (serum soluble transferrin receptor), TSAT (transferrin saturation). Multivariate models adjusted by age, sex, NYHA functional class, and LVEF.

**Table 4:** Multivariate linear regression models for 6MWT and MLHFQ according to LVEF.

	LVEF > 60% Multivariate Models			LVEF ≤ 60% Multivariate Models		
	β Coef.	*p* Value	Adj. R^2^	β Coef.	*p* Value	Adj. R^2^
**6 Min Walking Test (6MWT)**						
vs. iron deficiency (KDOQI)	−18.15	0.20	0.40	−37.31	0.023	0.33
vs. iron deficiency (FAIR−HF)	−14.54	0.24	0.40	−31.84	0.041	0.32
vs. anemia (WHO)	−7.48	0.56	0.40	−33.00	0.031	0.32
vs. TSAT < 20%	−16.53	0.23	0.40	−35.19	0.023	0.33
vs. sTfR (log)	10.13	0.54	0.39	−56.04	0.004	0.36
vs. ferritin (log)	−3.41	0.60	0.40	−5.86	0.47	0.31
vs. serum iron (log)	5.51	0.66	0.40	15.77	0.29	0.31
**Minnesota Living with Heart Failure Questionnaire (MLHFQ)**						
vs. iron deficiency (KDOQI)	6.80	0.037	0.15	2.77	0.41	0.05
vs. iron deficiency (FAIR−HF)	4.72	0.11	0.14	0.80	0.80	0.05
vs. anemia (WHO)	−0.38	0.89	0.13	2.99	0.33	0.05
vs. TSAT < 20%	5.30	0.09	0.14	1.07	0.74	0.05
vs. sTfR (log)	5.77	0.12	0.15	14.09	<0.001	0.13
vs. ferritin (log)	−0.79	0.60	0.13	2.16	0.18	0.05
vs. serum iron (log)	−1.76	0.55	0.13	−1.44	0.63	0.05

6MWT (6-Min Walking Test), adj. (adjusted), coef. (coefficient), FAIR-HF (Ferinject Assessment in patients with Iron deficiency and chronic Heart Failure study), KDOQI (Kidney Disease Outcomes Quality Initiative), LVEF (left ventricular ejection fraction), MLHFQ (Minnesota Living with Heart Failure Questionnaire), NYHA (New York Heart Association), sTfR (serum soluble transferrin receptor), TSAT (transferrin saturation). Multivariate models adjusted by age, sex, NYHA functional class, and LVEF.

## Supplementary Materials

The following are available online at https://www.mdpi.com/2077-0383/9/4/1199/s1, Table S1: MLHFQ individual items according to iron deficiency, Table S2: Baseline characteristics of HFpEF patients according to LVEF.
