# Supplementary material for: Iron Deficiency: Impact on Functional Capacity and Quality of Life in Heart Failure with Preserved Ejection Fraction
Source: J Clin Med. 2020 Apr 22;9(4):1199. doi: 10.3390/jcm9041199 (PMC7230551; doi:10.3390/jcm9041199)
Supplement: Supplementary file 1 [file jcm-09-01199-s001.pdf]

Journal of
Clinical Medicine

MDPI

Table S1. MLHFQ individual items according to iron deficiency.

	**Iron deficiency**	**No iron Deficiency**	***p* value**
Minnesota living with heart failure questionnaire (MLHFQ)	*N* = 325	*N* = 122	
Individual items (min: 0, max: 5)			
1. Swelling of legs	3.10 ± 2.06	2.86 ± 2.08	0.29
2. Having a rest during the day	3.73 ± 1.79	3.29 ± 2.03	0.036
3. Walking or climbing stairs	4.00 ± 1.64	3.66 ± 1.77	0.07
4. Housekeeping or gardening	3.43 ± 2.04	2.83 ± 2.14	0.008
5. Going away	3.62 ± 1.97	3.21 ± 2.06	0.06
6. Sleep	2.83 ± 2.09	2.38 ± 2.02	0.036
7. Friends or family	1.84 ± 2.01	1.39 ± 1.88	0.027
8. Earning profession	0.88 ± 1.75	0.68 ± 1.54	0.25
9. Hobbies	2.27 ± 2.09	1.75 ± 2.06	0.017
10. Sexual activity	1.27 ± 1.93	1.02 ± 1.80	0.19
11. Appetite	2.32 ± 2.03	2.11 ± 2.05	0.32
12. Feeling short of breath	3.68 ± 1.83	3.40 ± 1.94	0.17
13. Feeling fatigued	3.97 ± 1.61	3.78 ± 1.77	0.31
14. Stay in hospital	3.42 ± 2.09	3.06 ± 2.16	0.11
15. Cost of medical care	0.61 ± 1.36	0.48 ± 1.21	0.36
16. Side effects drugs	0.25 ± 0.84	0.29 ± 0.89	0.71
17. Burden for relatives	1.35 ± 1.72	1.20 ± 1.75	0.40
18. Loss of self-control	1.26 ± 1.73	1.16 ± 1.79	0.58
19. Worried	2.76 ± 1.89	2.43 ± 1.87	0.10
20. Concentration	1.12 ± 1.59	0.82 ± 1.42	0.06
21. Depression	1.66 ± 1.86	1.30 ± 1.62	0.041

Data presented as mean ± SD.

Table S2. Baseline characteristics of HFpEF patients according to LVEF.

	**LVEF >60%** *N* **= 220 (49%)**	**LVEF ≤60%** *N* **= 227 (51%)**	***p* value**
Age (years)	76.5 ± 9.2	75.0 ± 9.2	0.09
Female sex	151 (68.3)	113 (49.8)	<0.001
BMI (kg/m^2)	29.4 ± 6.3	28.8 ± 6.1	0.31
LVEF (%)	68.5 ± 5.5	56.0 ± 3.4	<0.001
Ischemic etiology of CHF	38 (17.0)	64 (27.5)	0.01
NYHA functional class			
I	24 (10.9)	23 (10.1)	0.91
II	89 (40.5)	104 (45.8)	0.29
III	88 (40.0)	78 (34.4)	0.26
IV	19 (8.6)	19 (8.4)	0.99
Comorbidities			
Hypertension	201 (90.1)	198 (85.0)	0.13
COPD	47 (21.4)	46 (20.3)	0.87
Diabetes mellitus	102 (55.7)	121 (51.9)	0.22
Chronic kidney disease	136 (61.0)	151 (64.8)	0.49
Anaemia	127 (57.0)	127 (54.5)	0.63
Iron deficiency (KDOQI)	166 (74.4)	159 (69.2)	0.24
Dependency	78 (35.0)	68 (29.2)	0.39
Medications			
ACEIs or ARBs	155 (69.5)	149 (63.9)	0.30
Beta-blockers	173 (77.6)	187 (80.3)	0.56
MRAs	17 (7.6)	35 (15.0)	0.019
Digoxin	35 (15.7)	39 (16.7)	0.86
Loop diuretics	208 (93.3)	212 (91.0)	0.46
Statins	119 (53.4)	133 (57.1)	0.48
Antiplatelets	72 (32.3)	86 (36.9)	0.35
Anticoagulants	133 (59.6)	133 (57.1)	0.65
Laboratory values			
Hemoglobin (g/dL)	12.0 ± 1.9	12.3 ± 1.9	0.16
eGFR (mL/min/1.73m^2)	55.4 ± 24.6	55.2 ± 23.0	0.95
Ferritin (ng/mL)	135 [67.8-260.5]	158 [72.5-290.5]	0.26
Transferrin (mg/dL)	253.9 ± 53.1	242.4 ± 49.5	0.018
Serum iron (pg/dL)	58.8 ± 28.4	64.7 ± 36.6	0.06
TSAT (%)	17.2 ± 8.7	20.0 ± 13.0	0.008
Ferritin index	0.99 ± 0.9	0.89 ± 0.5	0.14
sTfR (mg/L)	1.87 ± 0.8	1.83 ± 0.8	0.67
NT-proBNP (pg/mL)	1223 [595-2855]	1319.5 [715-3108]	0.39
C-reactive protein (mg/dL)	0.8 [0.3-1.8]	0.8 [0.3-2.1]	0.96
6MWT (meters walked)	274.3 ± 87.4	288.0 ± 109.3	0.24
MLHFQ, Overall summary score (min: 0, max: 105)	50.1 ± 22.0	45.8 ± 23.2	0.044

Data presented as mean ± SD, N (%) or median [interquartile range]. 6MWT (& minute walking test), ACEI (angiotensin converting enzyme inhibitor), ARB (angiotensin receptor blocker), BMI (body mass index), BP (blood pressure), CHF (chronic heart failure), COPD (chronic obstructive pulmonary disease), eGFR (estimated glomerular filtration rate), HFpEF (heart failure with preserved ejection fraction), ID (iron deficiency), LVEF (left ventricular ejection fraction), MLHFQ (Minnesota Living with Heart Failure Queastionnaire), MRA (mineralocorticoid receptor antagonist), NT-proBNP (N-terminal pro-B type natriuretic peptide), NYHA (New York Heart Association), sTfR (serum soluble transferrin receptor), TSAT (transferrin saturation). Dependency defined as Barthel test score <90 points.
